# Transforming undergraduate dental education: the impact of artificial intelligence

**DOI:** 10.1038/s41415-024-7788-7

**Published:** 2025-01-10

**Authors:** Molly Harte, Barbara Carey, Qingmei Joy Feng, Ali Alqarni, Rui Albuquerque

**Affiliations:** 41415215390001 Department of Oral Medicine, Guy´s and St Thomas´ NHS Foundation Trust, London, UK; 41415215390002 Department of Head and Neck Surgical Oncology, Guy´s and St Thomas´ NHS Foundation Trust, UK; 41415215390003 Department of Oral & Maxillofacial Surgery and Diagnostic Sciences, Faculty of Dentistry, Taif University, Taif 21944, UK; 41415215390004 Department of Oral Medicine, Guy´s and St Thomas´ NHS Foundation Trust, London, UK; Faculty of Dentistry, Oral and Craniofacial Sciences, King´s College London, London, UK

## Abstract

Artificial intelligence (AI) is a rapidly evolving area, having had a transformative effect within some areas of medicine and dentistry. In dentistry, AI systems are contributing to clinical decision-making, diagnostics and treatment planning. Ongoing advances in AI technology will lead to further expansion of its existing applications and more widespread use within the field of dentistry. The predicted transformation of current practice within dentistry brought about by AI necessitates the education of undergraduate dental students on the topic of AI. AI will act as a complementary tool to clinical expertise and skill, rather than a replacement. AI education frameworks should integrate with existing traditional dental education. It is important that dental students develop a sound understanding of the ethical considerations surrounding AI, ensuring its safe and responsible use. This article aims to consider the status of current undergraduate dental education and the possible impact of AI, examining both the challenges and opportunities, and the practicalities, of incorporating AI into existing undergraduate dental curricula. Understanding and envisioning the future landscape shaped by AI will allow for development of a future dental workforce who are skilled, confident and responsible AI users.

## Key points

- This paper provides an overview of the current use of artificial intelligence (AI) in dentistry.
- This paper examines the need for inclusion of AI in undergraduate dental training programmes, with focus on the importance of education around the ethical use of AI in dentistry.
- This paper provides recommendations for, while acknowledging the challenges of, incorporation of AI into dental undergraduate curricula.

## Introduction

Undergraduate dental education has historically followed a traditional curriculum split into two main areas: theoretical knowledge and clinical skills, with segregation of basic and clinical sciences. Over the last three decades, there has been gradual shift from more traditional teaching methodologies (eg lectures and rote learning) to incorporation of a problem-based learning style, allowing combined rather than segregated learning and fostering critical thinking skills needed for self-directed learning.^1^ Following successful implementation in several undergraduate medical education settings in the 1960s and 1970s, problem-based learning was first introduced in dental education in 1990 in The Faculty of Odontology in Malmö, Sweden.^2^ It continues to be integrated into undergraduate dental education programmes globally.

In addition to the shift to a problem-based learning style, technological advances have also changed the delivery of undergraduate dental clinical skills training. Phantom head training, where extracted teeth are mounted in a model to simulate a patient mouth, has been the cornerstone of clinical skills education in dental schools since the early 1900s.^3^ More recently, computer-supported, virtual reality (VR) and augmented reality simulators have been introduced to dental education, with several dental simulators available, providing a safe and supported learning environment in which students can develop the skills needed to detect and treat a variety of dental diseases.^4^ A major benefit of VR simulators for clinical skills teaching is the immediate and accurate feedback that can be provided by the software, allowing students to train independently, while also reducing the number of clinical supervisors required.^5^

Looking to the future of undergraduate dental education, artificial intelligence (AI) should be considered. AI is the capability of a computer system or machine to replicate normal human cognitive function. This can include image, speech and pattern recognition.^6^ Over the past decade, the rapid evolution of AI technologies has adapted several sectors. The growing use of AI technology into daily functions has started to transform our lifestyles and it is increasingly clear it will also transform professional practice.^7^ The potential benefits of AI to the fields of medicine and dentistry are numerous. In both, the accurate and timely diagnosis of disease is an essential clinical skill and an important step to formulate an appropriate treatment plan. Machine learning (an area of AI) can assist diagnosis through pattern recognition, improving accuracy and reducing clinical cost and time.^8^ Machine learning can also be used in clinical decision-making, forming a more reliable understanding of patient health and predict clinical outcomes, assisting clinicians with treatment decisions.^8^

In dentistry, the role of AI in diagnosis of diseases involving the teeth, periodontal tissues and jaw bones has been applied.^9^ The use of AI technology in dentistry will become more prevalent over the next decade. It is essential that undergraduate dental students are able to understand and use AI safely and ethically.

This article aims to illustrate the potential influence of AI on undergraduate dental education and provide recommendations for the safe, effective and ethical incorporation of AI into the undergraduate dental curricula.

## Applications of AI in dentistry

In clinical dentistry, one area of AI with evidenced potential and expanding use is machine learning (ML). In ML, large data sets are used to teach algorithms, imitating human learning, and gradually improving in accuracy over time.^10^ Neural networks (NNs) are a subcategory of ML of relevance to dentistry. An NN is a non-linear mathematical model, inspired by the human neuron, where artificial neurons connect with each other, each with a threshold value that must be met before passing data along to the next artificial neuron.^11^ In dentistry, NNs have been engineered to solve the specific image classification task of determining whether a radiographic image demonstrates a carious tooth or not.^12^

Broadly speaking, NNs can be used to detect, segment and classify structures. In an NN used to detect dental caries, the system will analyse an image to detect the presence and shape of teeth before identifying the exact location of dental caries and labelling this accordingly.^13^ The use of NNs in dentistry has a wide range of potential applications, not limited to the detection of dental caries, but also in the detection of periapical pathology, periodontal disease and diseases of the jaw bones.^9^ The use of NNs for interpretation of diagnostic imaging is an exciting prospect with the potential to overcome both intra- and inter-clinician variance, therefore improving quality and standardisation of care.^13^ At present, NNs do not negate the need for skilled diagnosticians but can be thought of as diagnostic-assistance tools, allowing faster and more systematic evaluation of diagnostic dental imaging.^13^

Chatbots are being adopted in dentistry to help patients make appointments and answer patient queries but are useful tools for patient and undergraduate education.^14^^,^^15^ Natural language processing - the ability of a computer system to understand, interpret and manipulate human language - has been suggested as a clinical notes mining tool (a mechanism for identification of certain diseases from analysis of the patient's electronic health records).^16^

Another transformative subcategory of AI includes robotics. Its uses include robotic articulation of teeth, assisted robotic implant placement and robotic orthognathic surgery, but could extend to clinical training with robotic haptic virtual reality simulators.^17^

## AI in dental education: practical and theoretical uses

As AI has a number of developing and future potential applications in dentistry, it is important that dental students are educated on its use.^18^ It should be made apparent at an early stage in dental training that AI technologies will not replace clinical skills and the need for clinical expertise but is an adjuvant tool, particularly in diagnostics.^19^ While students will need to be knowledgeable and confident in the incorporation of AI technologies to their practice, dental curricula should continue to encourage critical thinking and problem solving.^20^

In addition to the above, conventional teaching modalities, including lectures and seminars, and learning strategies, such as memorisation and group revision, can be enhanced by AI in a dental education setting.

The final, and perhaps most difficult to navigate, aspect of AI in undergraduate education is the use of text-generative AI programmes. These carry huge benefits in terms of teaching assistance, supporting learning and aiding creativity,^21^ but they also raise the issue of plagiarism. ChatGPT by OpenAI is an increasingly popular AI tool with text-generative capability and the ability to provide human-like responses. ChatGPT may be used to generate essays and question answers and users may falsely submit AI-generated text as their own work.^22^ Freely accessibly tools like ChatGPT highlight the need for AI detection software to be incorporated into existing anti-plagiarism tools. As well as educating undergraduate students on the ethical use of AI as part of its direct applications to dentistry, the ethical use of AI more broadly (misuse and responsibility) is also a key topic for educators.^20^

## Incorporating AI into the dental undergraduate curriculum

The growing use of AI in dentistry necessitates adequate translation into undergraduate dental curricula. One of the difficulties when considering how to introduce AI into an undergraduate curriculum is defining what students need to know, particularly with the current rate of advancement of AI technologies.^21^ Although AI has multiple potential applications in dentistry, it is currently not widely used. Therefore, those involved in curriculum design need to predict the level of AI exposure students are likely to have by the time they qualify in order to define the level of knowledge they will need to practise safely.

AI is not the only aspect of dentistry where there is constant evolution. Dental schools cannot be expected to provide training that will see newly qualified dentists through their entire careers without the need to undertake additional training and continued professional development. However, it is important that the fundamentals of AI are introduced to dentists early in their careers, ideally as undergraduates, in order to ensure clinicians can continue to adapt to new AI technologies and applications as they emerge.^23^ Of particular importance is students having a clear understanding of the ethical principles of AI use. On consideration, a balanced AI curriculum for undergraduate dental students should cover several key areas, outlined below.

### Basic terminology

As discussed above, multiple categories of AI exist. In order for students to better understand the technologies they are using, they should be introduced to the fundamental concepts of AI and the terminology associated with this. This would include being able to define and understand terms like ML, deep learning, NNs and algorithms.

### Principles and applications

An understanding of the core principles of AI, including data-driven decision-making and pattern recognition, is essential to allow students to recognise how the systems they use work. This will allow deeper understanding of how AI systems can be applied within dentistry, while also appreciating where there may be potential shortcomings within an AI system.

### Current use of AI in dentistry

Although it is challenging to predict exactly how the integration of AI with dentistry will look in the future, any undergraduate curriculum should provide an overview of the current use of AI in dentistry. This may be supplemented by real-life examples of current AI use, both with success stories and cautionary tales, providing more relevance to the teaching and better engagement from students.

### Ethical use of AI

Ethical use of AI is a key message that should be reiterated to undergraduate dental students throughout their training. This should cover topics such as patient privacy, data security, informed consent and responsible AI use. Training in ethics surrounding AI at an early stage in their training will ensure the future generation of dentists are able to maintain high ethical standards when using AI throughout their careers.

### Limitations of AI

Although the benefits and potential applications of AI should be understood, students also need to be aware of the limitations and challenges associated with AI use in dentistry. They must be acutely aware of the need for human oversight.

## Emphasising ethical implications

As with the introduction of any new technology which promises change across both personal and professional sectors, fear exists about the growing use of AI. Concerns include the risk of job jeopardisation, reduced human accountability, harmful misuse and discrimination. The need for assured ethical use of AI, particularly when used in healthcare settings, is essential. Over 80 guidelines for the ethical use of AI have been developed globally,^24^ and although there is no global consensus on the ethical principles that should be applied to AI use within healthcare, there is convergence on five main principles: transparency; justice and fairness; non-maleficence; responsibility; and privacy.^24^ The Montreal Declaration^25^ set out an ethical framework for the development and deployment of AI, with ten key principles summarised in Table 1 which can be easily applied to AI use in dentistry and healthcare settings more broadly.

**Table 1:** Ethical framework for the development and deployment of AI^25^

1	Wellbeing	AI systems should support individuals and allow people to use their mental and physical capacity. AI systems should not contribute to stress or anxiety
2	Respect for autonomy	AI systems should allow individuals to fulfil their own objectives. AI systems should not impose lifestyles on individuals or encourage dependency
3	Protection of privacy and intimacy	Every individual must have control over their personal data when considering collection, use and dissemination. AI systems must guarantee data confidentiality
4	Solidarity	AI systems must allow human collaboration and support human relationships. Healthcare settings where AI systems are used must be aware of the importance of patient relationships with family and staff. AI systems must not be implemented to replace people in roles where human relationships are required
5	Democratic participation	AI systems that make decisions affecting a person's life or quality of life must be intelligible to their creators. Any person using a service should know if they are interacting with an AI system or a real person and should know if a decision affecting them was made by an AI system
6	Equity	AI systems must be designed and trained not to discriminate based on protected characteristics. AI systems should produce social and economic benefits for all groups
7	Diversity inclusion	AI systems must allow maintenance of societal and cultural diversity. AI systems must reflect the diversity of individuals and must not be developed to limit free expression of ideas
8	Prudence	Those involved in development of AI systems must be aware of the possible adverse consequences of AI systems and must take appropriate action to limit harmful use. Where an AI system is misused and endangers public health or safety, access to the algorithm should be restricted
9	Responsibility	AI systems must not lead to reduced human responsibility with regard to decisions being made. Only humans can be accountable for decisions based on recommendations made by AI systems. Those who authorise an AI system to commit a crime or offense are responsible for that crime or offense
10	Sustainable development	AI systems must aim for energy efficiency and minimise our impact on ecosystems and biodiversity. Public and private sectors must support environmentally responsible development of AI systems

The inclusion of education on ethical implications of AI as part of the dental undergraduate curriculum ensures students possess not only technical knowledge of AI but also a deeper understanding of the moral issues that govern its safe application.

## Practical limitations of a curriculum change

Changes to the medical and dental undergraduate curricula are subject to strict review and quality appraisal. In the UK, the General Dental Council is responsible for quality assurance of dental education and training programmes and has developed a set of standards that education providers must meet.^26^ Any alterations to undergraduate curricula at a local level, therefore, must be carefully considered before implementation. A further challenge is the addition of new material to an already-full curriculum. For AI to be incorporated into an undergraduate dental curriculum, there must be substantial evidence that this is a beneficial topic for students to learn about and agreement that this is a sufficiently important area to justify its priority over other areas of the existing dental curriculum.

## Recommendations

- The traditional undergraduate dental curriculum must transition to the age of AI
- Undergraduate dental students must appreciate the limitations of AI and AI education should be delivered in a complementary fashion to existing dental education
- The ethical implications of AI need to be at the heart of AI education to encourage responsible and safe use of these developing technologies.

Given the rapid evolution of AI technologies, defining what students should know is complex. Curricula should aim to cover the fundamental terminology and principles, current applications and ethical considerations, and provide students with an awareness of the limitations and challenges associated with AI use. The ethical issues associated with AI use should be a key aspect of any AI curriculum. Incorporating this into an undergraduate dental curriculum will provide the best foundation for new dentists to prioritise patient safety, uphold ethical standards and promote the responsible use of AI within their field.

## Data Availability

Data availability is not applicable to this article as no new data were created or analysed in this study.
